# Measurement of mechanical withdrawal thresholds and gait analysis using the CatWalk method in a nucleus pulposus-applied rodent model

**DOI:** 10.1186/s40634-017-0105-5

**Published:** 2017-09-29

**Authors:** Takuya Kameda, Yoichi Kaneuchi, Miho Sekiguchi, Shin-ichi Konno

**Affiliations:** Department of Orthopaedic Surgery, Fukushima Medical University School of Medicine, 1 Hikarigaoka, Fukushima City, 960-1295 Japan

**Keywords:** Nucleus pulposus, Lumbar disc herniation, Dorsal root ganglion, Mechanical withdrawal threshold, Gait analysis, CatWalk

## Abstract

**Background:**

There are some previous reports of gait analysis using a rodent pain model. Applying the CatWalk method, objective measurements of pain-related behavior could be evaluated, but this method has not been investigated using the nucleus pulposus (NP) applied model, which was developed as a model of lumber disc herniation. We aimed to measure mechanical withdrawal thresholds and analyze gait patterns using the CatWalk method for the evaluation of the pain-related behavior caused by NP application.

**Methods:**

Twenty-four nine-week-old female Sprague-Dawley rats were randomly divided into two experimental groups, the NP group (*n* = 12), in which autologous NP from the tail was applied to the left L5 dorsal root ganglion, and the sham-operated group (*n* = 12). Measurements of mechanical withdrawal thresholds were performed using von Frey filaments touching the left footpads, and gait analysis was performed using the CatWalk method. These experiments were conducted 1 day before surgery and 7, 14, 21, and 28 days after surgery. Data were statistically analyzed using the Wilcoxon rank-sum test.

**Results:**

The NP group showed significantly lower withdrawal thresholds than the sham group at days 14 and 21. *Stand* (duration of contact of a paw with the glass plate) was significantly higher in the NP group at days 7 and 14, whereas *step cycle* (duration between two consecutive initial contacts of the same paw) and *duty cycle* (*stand* as a percentage of *step cycle*) were the same at day 7. Long *initial dual stance* (duration of ground contact for both hind paws simultaneously, but the first one in a *step cycle* of a target hind paw) of the right hind paw was measured at days 7 and 14. The left hind paw per right hind paw ratio of the *stand index* (speed at which the paw loses contact with the glass plate) and *mean intensity* (mean intensity of the complete paw) changed at day 7 or 14. *Phase dispersion* (parameter describing the temporal relationship between placement of two paws) of the hind paws decreased at day 7.

**Conclusions:**

Rats with applied NP showed a decreased withdrawal threshold and abnormal gait. The differences in gait parameters between the NP and sham groups were observed at an earlier time point than the withdrawal thresholds. Gait analysis could be an effective method for understanding pain caused by applied NP.

## Background

Lumbar disc herniation is a major cause of sciatica, and mechanical and chemical factors are involved in its pathology. To evaluate the effects of various proinflammatory cytokines (Kawakami et al. 1996; Olmarker and Larsson 1998; Igarashi et al. 2000; Sasaki et al. 2007) and other factors (Brisby et al. 2000; Hashizume et al. 2007; Otoshi et al. 2010; Uesugi et al. 2011), pain-related behavior in a nucleus pulposus (NP) rat model has previously been evaluated using the von Frey method (Igarashi et al. 2000; Hashizume et al. 2007; Kato et al. 2008; Otoshi et al. 2011; Uesugi et al. 2011). This autologous NP applied rat model was developed to determine the pathological changes associated with lumber disc herniation (Yabuki et al. 1998). The advantage of applying this model is to enable investigation of the NP-initiated pathological changes that more closely resemble the clinical condition.

Recently, a large variety of behavioral research using rodent spinal pain models has been reported. Measurement of mechanical withdrawal thresholds or thermal stimulation of the foot surface has often been used, with the former indicating mechanical allodynia and the latter revealing thermal hyperalgesia. With respect to von Frey testing, there are some limitations. For example, it is known that lower extremity hypoalgesia is clinically observed in humans (Ljunggren 1983) when the entrapment of spinal nerves occurs in lumbar disease (Saegusa 1983). However, in rodents, a low withdrawal threshold has been observed, and it reflects mechanical allodynia, not hypoalgesia.

Gait analysis has been examined in several rodent models, not only in the pain model. CatWalk (Hamers et al. 2001), DigiGait, TreadScan (Dorman et al. 2014) and Dynamic Weight Bearing Test (Robinson et al. 2012) are the common devices used for rodent gait analysis (Lakes and Allen 2016). The CatWalk method allows easy quantitation of a large number of locomotion parameters during walkway crossing (Hamers et al. 2001), and one of the advantages of this method is that it reveals the details of a more voluntary gait compared to a device with a treadmill. It was reported that gait parameters change due to radiculopathy (Vrinten and Hamers 2003) and intervertebral disc injury (Miyagi et al. 2013), and the CatWalk method might be useful for researching the relationship between spinal pain and gait using rodent models. However, gait analysis using the CatWalk method has not been explored in the NP model. Thus, we hypothesized that the details of gate abnormalities initiated by NP application, which can mimic the pathology of lumber disc herniation, could be revealed using the CatWalk method.

The aim of this study was to investigate pain-related behavior initiated by NP application using measurement of mechanical withdrawal thresholds and gait pattern analysis using the CatWalk method.

## Methods

### Animals

A total of 24 nine-week-old female Sprague-Dawley rats (Japan SLC, Shizuoka, Japan) weighing 190 to 210 g were used in this study. The rats were housed in plastic cages at room temperature (21–24 °C) in a 12-h light and dark cycle with free access to food and water. The animal experiment was carried out under the guidance of the Animal Care and Use Committee in accordance with Guidelines for Animal Experiments of our institution and the Japanese Government Law Concerning the Protection and Control of Animals.

### Experimental groups

The animals were divided into two experimental groups, the autologous NP application group (NP group: *n* = 12) and the sham-operated group (sham group: n = 12). The non-labeled cages which included 3 rat randomly separated into each experimental group.

### Surgical procedure

A combination of 0.3 mL of medetomidine hydrochloride (1.0 mg/mL; Nippon Zenyaku Kogyo, Fukushima, Japan), 0.8 mL of midazolam (5.0 mg/mL; Astellas Pharma, Tokyo, Japan), and 1.0 mL of butorphanol tartrate (5.0 mg/mL; Meiji Seika Parma, Tokyo, Japan) was prepared as an anesthetic. Before surgery, the animals were anesthetized by intraperitoneal injection of 0.1 mL/100 g of body weight of the above-described mixed anesthetic. They were then placed in the prone position, and an incision was made at the middle of the spine at the L4–L6 level. The fascia was incised, and the multifidus muscles were moved laterally to expose the facet joint between the left fifth and sixth lumbar vertebrae using an operating microscope. The left L5 dorsal root ganglion (DRG) was exposed after left L5/L6 facetectomy. By puncturing the 4/5 or 5/6 coccygeal vertebral discs with forceps after dorsal skin incision, autologous NP was harvested and applied to the left L5 DRG (Otoshi et al. 2010, 2011; Miyoshi et al. 2011; Sasaki et al. 2011; Sekiguchi et al. 2011). Fascia was closed with 4–0 nylon, and the skin was closed with surgical staples. Animals in the sham group underwent the same surgical procedure with the NP harvesting but without NP application to the DRG. All animals were returned to their original cages and monitored visually at the appropriate intervals to confirm recovery from anesthesia and their ability to access food and water by themselves.

### Mechanical withdrawal thresholds

Behavioral testing of mechanical withdrawal thresholds using von Frey filaments was performed 1 day before surgery to serve as a baseline and 7, 14, 21, and 28 days after surgery. The left hind paw withdrawal response to von Frey filament (Aesthesio®, DanMic Global LCC, San Jose, CA, USA) stimulation of the plantar surface of the footpads was determined. The rats were placed individually in an acrylic cage with a mesh floor and allowed to acclimatize until cage exploration and major grooming activities ceased. The lateral-plantar surface of the operated (left) hind paw was stimulated with 9 von Frey filaments (1.0, 1.4, 2.0, 4.0, 6.0, 8.0, 10.0, 15.0, and 26.0 g, as rated by the manufacturer) threaded under the mesh floor. Stimulation was initiated with the 1.0-g filament and applied just until the filament bent for 2–3 s. If the rat did not withdraw its foot after stimulation, testing with the next stiffer filament was carried out in the same manner. The response was considered positive if the hind limb was lifted as an escape response. A single set of these procedures was performed to determine the threshold. When the rat did not show any escape response, the threshold was estimated to be 26 g considering the sensitivity limit of this experiment.

### Gait analysis

To evaluate the detailed functional changes in gait, gait analysis using the CatWalk method was performed (Hamers et al. 2001) using the CatWalk™ XT (Noldus Information Technology, Wageningen, The Netherlands). Animals crossed the vitreous corridor, and the floor was monitored by a charge-coupled device (CCD) camera located under the floor. Fluorescent light illuminating the corridor enabled the camera to capture footprint images. The light intensity reflected the force exerted by the paw. The images of real-time footprints were recorded in a computer, after which the parameters listed in Table 1 were calculated using the CatWalk XT software package (Noldus Information Technology, Wageningen, The Netherlands). Animals were trained on a CatWalk runway to make some uninterrupted runs 1 day prior to surgery in order to reduce stress. The actual trial was repeated until three consecutive uninterrupted runs were recorded. Rats visually observed to exhibit prolonged stopping or that turned backward in the runway were considered to have failed the trial. Positive or negative reinforcement to achieve more voluntary walking was not applied. All trials and measurements of mechanical withdrawal thresholds were performed on the same day.

**Table 1:** Overview of Analyzed Gait Parameters

Parameters		Explanation
Basic	*Average speed* (cm/s)	Speed of the animal’s body in the recorded run
	*Number of steps*	Total number of selected steps in one run
	*Body speed* (cm/s)	Speed calculated by dividing the distance that the animal’s body traveled from one initial contact of one paw to the next by the time to travel that distance
	*Body speed validation* (%)	Percentage calculated by dividing the absolute difference between the body speed and the average speed of a run by the average speed
Calculated from walking durations	*Stand* (s)	Duration of contact of a paw with the glass plate (Fig. 1)
	*Swing* (s)	Duration of no contact of a paw with the glass plate (Fig. 1)
	*Step cycle* (s)	Time in seconds between two consecutive initial contacts of the same paw: *stand* + *swing* (Fig. 1)
	*Duty cycle* (%)	*Stand* as a percentage of *step cycle*: *stand* / (*stand* + *swing*) × 100 (Fig. 1)
Calculated from bilateral paw durations	*Initial dual stance* (s)	The duration of ground contact for both hind paws simultaneously, but the first one in a step cycle of a target hind paw (Fig. 1)
	*Terminal dual stance* (s)	The duration of ground contact for both hind paws simultaneously, but the latter one in a step cycle of a target hind paw (Fig. 1)
	*Single stance* (s)	Duration in seconds of ground contact for a single hind paw
Calculated from the data of maximum contact	*Stand index*	Speed at which the paw loses contact with the glass plate
	*Max contact at* (%)	Max contact at (s) relative to *stand* of a paw Max contact at (s): the time in seconds since the start of the run that a paw makes maximum contact with the glass plate
	*Max contact area* (cm^2^)	Maximum area of a paw that comes into contact with the glass plate
Calculated from the light intensity data	*Max contact max intensity*	Maximum intensity at maximum contact of a paw
	*Max contact mean intensity*	Mean intensity of a paw at maximum contact
	*Max intensity at* (%)	Max intensity at (s) relative to *stand* of a paw Max intensity at (s): time in seconds since the start of the run that the maximum intensity is measured
	*Max intensity*	Maximum intensity of the complete paw
	*Min intensity*	Minimum intensity of the complete paw
	*Mean intensity*	Mean intensity of the complete paw
Calculated from the data of the complete paw print	*Print length* (cm)	Length (horizontal direction) of the complete print
	*Print width* (cm)	Width (vertical direction) of the complete paw print
	*Print area* (cm^2^)	Surface area of the complete print
	*Swing speed* (cm/s)	Speed of the paw during *swing*: *stride length* / *swing*
	*Stride length* (cm)	Distance between successive placements of the same paw
Other	*Regularity index* (%)	Number of normal step sequence patterns relative to the total number of paw placements
	*Base of support* (cm)	Average width between either the front paws or the hind paws (base of support of hind paws was analyzed in this study)
	*Sciatic functional index*	Measure of the functional recovery of the sciatic nerve that innervates the hind paws
	*Phase dispersions*	Parameters describing the temporal relationship between placement of two paws within a step cycle: ((Initial contact of target paw – Initial contact of anchor paw) / step cycle of anchor paw) × 100 (Fig. 1) Phase dispersions of two girdle pairs (right forepaw and left forepaw: LF-RF, right hind paw and left hind paw: LH-RH) were analyzed Left paws were always set as the anchor paw

**Fig. 1:**
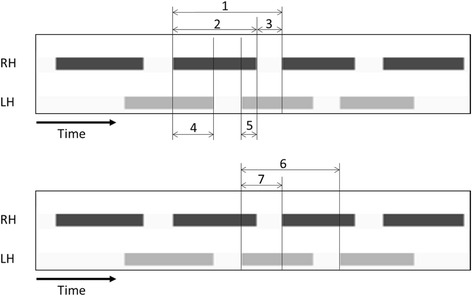
Timing of RH and LH placement during CatWalk runs with a graphical depiction of stance parameters. The X axis shows time, and the two different bands indicate the contact durations of the right hind paw (RH) and left hind paw (LH) with the ground. 1: *Step cycle* of RH. 2: *Stand* of RH. 3: *Swing* of RH. 2/1: *Duty cycle* of RH. 4: *Initial dual stance* of RH and *terminal dual stance* of LH. 5: *Terminal dual stance* of RH and *initial dual stance* of LH. 7/6: Phase dispersion of LH-RH

The 29 parameters listed in Table 1 were analyzed in this study. Initially, *average speed* and *number of steps* were examined as basic parameters. A total of 23 paw parameters based on individual paw prints, *regularity index*, *base of support*, *sciatic functional index* (de Medinaceli et al. 1982), and *phase dispersions* were calculated (Table 1). The right hind paw (RH) and left hind paw (LH) were analyzed for each animal. The mean values of paw parameters of RH and LH from three runs per animal were used for further analysis. To observe the overall effect on hind paws, RH and LH values from each run were combined and evaluated as the value of both hind paws. For parameters reflecting unilateral changes, we calculated the ratio of operated/healthy side of hind-paw parameters (LH/RH) for each run, and calculated the mean values in the same manner. Timing of RH and LH placement during CatWalk runs with a graphical depiction of the stance parameters are shown in Fig. 1.

### Statistical analysis

All measured values presented in the figures are expressed as the mean and standard error (shown as error bars). Parameters of the NP and sham groups were compared at each time point. According to the Shapiro–Wilk test, some experimental data were not distributed normally. Thus, statistical analysis was performed using the Wilcoxon rank-sum test in JMP® for Windows version 10.0.2 (SAS Institute Inc., Cary, NC, USA). A *p* value less than 0.05 was considered significant.

## Results

### Measurement of mechanical withdrawal threshold

**Fig. 2:**
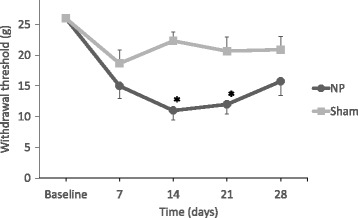
Changes in the mechanical withdrawal threshold of the footpad in rats. In the NP group, there are significant differences in the thresholds compared with the sham group on days 14 and 21 (*p < 0.05)

There was no significant difference in the withdrawal threshold between the two groups at baseline. At days 14 and 21, the animals in the NP group showed significantly lower withdrawal thresholds than those in the sham group (*p* < 0.05) (Fig. 2).

### Gait analysis using the CatWalk method

Eleven of the 29 parameters showed significant differences in hind-paw values between the NP and sham groups at one or more time points (*p* < 0.05, Table 2). Basic parameters (*average speed*, *number of steps* in one run) were not significantly different between the two groups at any of the time points (Table 2).

**Table 2:** Raw *P* values (Wilcoxon rank-sum test) for 30 different gait parameters (NP group versus sham group)

	LH				RH				Both hind paws			
	Day 7	Day 14	Day 21	Day 28	Day 7	Day 14	Day 21	Day 28	Day 7	Day 14	Day 21	Day 28
*Average speed*									NS	NS	NS	NS
*Number of steps*									NS	NS	NS	NS
*Body speed*	NS	NS	NS	NS	NS	NS	NS	NS	NS	NS	NS	NS
*Body speed validation*	NS	NS	NS	NS	NS	NS	NS	NS	NS	NS	NS	NS
*Stand*	NS	NS	NS	NS	NS	NS	NS	NS	0.0180	0.0480	NS	NS
*Swing*	NS	NS	NS	NS	NS	NS	NS	NS	NS	NS	NS	NS
*Step cycle*	NS	NS	NS	NS	NS	NS	NS	NS	0.0342	NS	NS	NS
*Duty cycle*	0.0324	NS	NS	NS	0.0236	NS	NS	NS	0.0018	NS	NS	NS
*Initial dual stance*	NS	NS	NS	NS	0.0052	0.0487	NS	NS	0.0150	NS	NS	NS
*Terminal dual stance*	0.0426	NS	NS	NS	NS	NS	NS	NS	0.0240	NS	NS	NS
*Single stance*	NS	NS	NS	NS	NS	NS	NS	NS	NS	NS	NS	NS
*Stand index*	NS	NS	NS	NS	NS	NS	NS	NS	NS	NS	NS	NS
*Max contact at*	0.0027	NS	NS	NS	NS	NS	NS	NS	0.0037	NS	NS	NS
*Max contact area*	NS	NS	NS	NS	NS	NS	NS	NS	NS	NS	NS	0.0223
*Max contact max intensity*	NS	NS	NS	NS	NS	NS	NS	NS	NS	NS	NS	NS
*Max contact mean intensity*	NS	NS	NS	NS	NS	NS	NS	NS	NS	NS	NS	NS
*Max intensity at*	NS	NS	0.0027	0.0229	NS	NS	NS	NS	NS	NS	NS	NS
*Max intensity*	NS	NS	NS	NS	NS	NS	NS	NS	NS	NS	NS	NS
*Min intensity*	NS	NS	NS	NS	NS	NS	NS	NS	NS	0.0420	NS	NS
*Mean intensity*	NS	NS	NS	NS	NS	NS	NS	NS	NS	NS	NS	NS
*Print length*	NS	NS	NS	NS	NS	NS	NS	NS	NS	NS	NS	NS
*Print width*	NS	NS	NS	NS	NS	NS	NS	NS	NS	NS	NS	NS
*Print area*	NS	NS	NS	NS	NS	NS	NS	NS	NS	NS	NS	0.0129
*Swing speed*	NS	NS	NS	NS	NS	NS	NS	NS	NS	NS	NS	NS
*Stride length*	NS	NS	NS	NS	NS	NS	NS	NS	NS	NS	NS	NS
*Regularity index*									NS	NS	NS	NS
*Base of support (hind)*									NS	NS	NS	NS
*Sciatic functional index*									NS	NS	NS	NS
*Phase dispersions (RH-LH)*									0.0481	NS	NS	NS
*Phase dispersions (LF-RF)*									NS	NS	NS	NS
*Phase dispersions (RF-LH)*									NS	NS	0.0003	NS
*Phase dispersions (LF-RH)*									NS	NS	NS	NS

*NS*: not significant

#### Walking duration

**Fig. 3:**
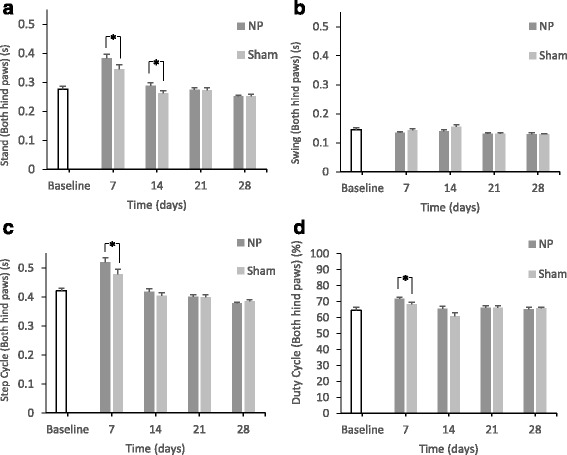
Changes in the paw statistics related to walking duration for both hind paws. In the NP group, there are significant differences in *stand* compared with the sham group on days 7 and 14 (*p < 0.05) (A). There are no significant differences in *swing* compared with the sham group at any time points (B). There are significant differences in the *step cycle* compared with the sham group on day 7 (*p < 0.05) (C). There are significant differences in the *duty cycle* compared with the sham group on day 7 (*p < 0.05) (D)

At days 7 and 14, the duration of *stand* was longer in the NP group than in the sham group (*p* < 0.05) (Fig. 3a). *Step cycle* was longer and *duty cycle* was greater in the NP group than in the sham group at day 7 (p < 0.05), as shown in Fig. 3c and d, respectively. *Swing* was not significantly different between the two groups at any of the time points (Fig. 3b).

#### Bilateral paw duration

**Fig. 4:**
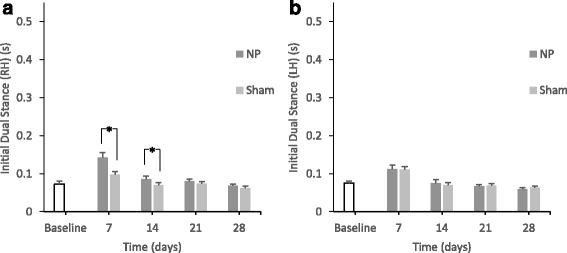
Changes in the paw statistics calculated from bilateral paw duration (*p < 0.05). In the NP group, there are significant differences in RH *initial dual stance* compared with the sham group on days 7 and 14 (*p < 0.05) (A). There are no significant differences in LH *initial dual stance* compared with the sham group (B)

At days 7 and 14, there were significant differences in RH *initial dual stance* between the two groups (*p* < 0.05) (Fig. 4a). There were no significant differences in LH *initial dual stance* between the two groups (Fig. 4b).

#### Parameters calculated from the data of maximum contact and light intensity

**Fig. 5:**
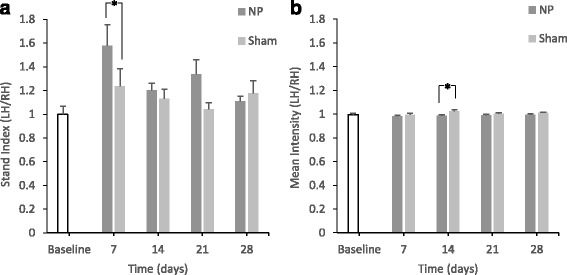
Changes in the parameters calculated from the data of maximum contact and light intensity (*p < 0.05) (A) In the NP group, there are significant differences in the *stand index* compared with the sham group on day 7 (*p < 0.05). There are significant differences in *mean intensity* compared with the sham group on day 14 (*p < 0.05) (B).

At day 7, *stand index* was significantly different between the two groups (p < 0.05) (Fig. 5a). At day 14, there was a significant difference in *mean intensity* between the two groups (*p* < 0.05) (Fig. 5b).

#### Other parameters

**Fig. 6:**
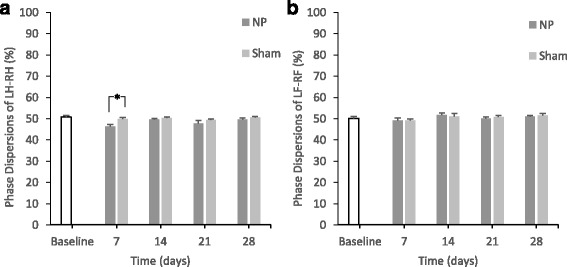
Changes in *phase dispersions* (*p < 0.05) In the NP group, there are significant differences in *phase dispersions* of LH-RH compared with the sham group on day 7 (*p < 0.05) (A). There are no significant differences in *phase dispersions* of LF-RF (B).

There were no significant differences in *regularity index*, *base of support*, and *sciatic functional index* between the two groups (Table 2). Concerning *phase dispersions*, there were significant differences in LH-RH between the two groups at day 7 (*p* < 0.05) (Fig. 6a). In contrast, there were no significant differences in LF-RF (right forepaw and left forepaw) *phase dispersions* (Fig. 6b).

## Discussion

In the present study, abnormal gait as a pain-related behavior was observed objectively and evaluated from multiple perspectives. Significant differences in the mechanical withdrawal thresholds between the two groups were observed at days 14 and 21. Furthermore, using the CatWalk method, there were significant differences in *stand*, *step cycle*, *duty cycle*, *dual stance*, *stand index*, *mean intensity*, and *phase dispersion* at days 7 and/or 14.

*Stand* is mentioned frequently in previous research on gait analysis, and not only in studies utilizing the CatWalk method (Hamers et al. 2001; Tang et al. 2009; Allen et al. 2011). Accordingly, this is the most basic parameter for gait analysis. In this study, *stand*, *step cycle*, and *duty cycle* changed at an early time point. These parameters indicate the duration of contact of a paw with the glass plate, the duration of the walking cycle, and the contacting duration as a percentage of the gait cycle. Additionally, there were no significant differences in *swing*, which corresponds to the duration of no contact. These results indicate that the slower cycle of gait in the NP group is primarily the result of *stand*, not *swing*. In previous studies using a chronic constriction injury (CCI) model (Vrinten and Hamers 2003) and a crushed sciatic nerve model (Bozkurt et al. 2008), *stand* decreased. In another study using an intervertebral disc injury model (Miyagi et al. 2013), *stand* increased. The NP model showed different changes from other unilateral neuropathic pain models. In addition, the present study revealed that the differences in these parameters between the two groups disappeared by 14 days after surgery. The NP applied rat model shows a shorter decrease in the withdrawal threshold (Uesugi et al. 2011; Miyoshi et al. 2011) compared to the CCI model (Vrinten and Hamers 2003) even after taking the differences in study design into consideration. Thus, the NP model could have a shorter pain duration and milder damage, which is closer to the clinical condition of lumber disc herniation. It is assumed that a change in gait that more closely resembles clinical pathology was achieved in the present study.

*Dual stance* was analyzed in the present study. As shown in Fig. 1, since the *initial dual stance* of RH means the duration from the beginning of the contact of RH to the following ending of the contact of LH, the long *initial dual stance* of RH reflects early contact of RH and late contact of LH if the *initial dual stance* of LH, i.e., the opposite side, was the same for the two groups. In the present study, the *initial dual stance* of RH in the NP group was longer than that in the sham group. Furthermore, there was no difference in the *initial dual stance* of LH between the two groups. Therefore, the present results indicate that animals in the NP group made contact with the floor using the paw of the non-operated side earlier than the sham animals. It is assumed that the animals avoided contact of a single painful LH with the floor. To the best of our knowledge, there are no previous reports of dual stance caused by unilateral pain. *Dual stance* could be an important parameter for evaluating the abnormal asymmetric rhythm of gait. In other words, *dual stance* in the present study indicates limping caused by NP application and could therefore be an important parameter for evaluating the abnormal asymmetric rhythm of gait.

The *stand index* is the speed of raising the paw from the time point of maximum contact. Therefore, a larger *stand index* ratio (LH/RH) corresponds to a higher speed of raising the left paw than the right paw. The results suggested that animals in the NP group tended to raise the paw of the operated side more quickly. The *stand index* has often been mentioned in evaluations of models of brain injury (Mountney et al. 2013) and of neurological diseases such as Huntington’s disease (Vandeputte et al. 2010) and amyotrophic lateral sclerosis (Mead et al. 2011; Vergouts et al. 2015). However, the results varied by model, and the evaluation of pain through changes in the *stand index* requires further study.

Concerning the *mean intensity*, a low LH/RH ratio indicates that the force due to the paw of the operated side was smaller than that of the non-operated side. Therefore, this result suggests that animals in the NP group tended to avoid putting weight on the paw of the operated side. Although we should be careful when interpreting this result since almost all mean values for the NP group were close to 1. In past studies using the CCI model and the crushed sciatic nerve model, the *mean intensity* decreased (Vrinten and Hamers 2003; Bozkurt et al. 2008). The value of *mean intensity* obtained in this study agrees with the results obtained using those models. Furthermore, the value of *mean intensity* may reflect allodynia (Vrinten and Hamers 2003).

Girdle pairs (RF-LF, RH-LH) usually yield a *phase dispersion* value of 50% when the animal walks at a moderate speed. The *phase dispersion* value indicates inter-paw coordination (Kloos et al. 2005). In the present study, there were significant differences in RH-LH between the NP and sham groups. This indicates that coordination of each hind paw showed different patterns, whereas the forepaw showed good paw coordination. The *phase dispersions* of girdle pairs could be indices for limping. Although the pain continued to day 21 according to the von Frey test, limping caused by NP application was indicated by phase dispersion until day 7. Finally, regarding the limping, or asymmetric abnormality of the gait, *dual stance* and *phase dispersion* were defined as the parameters to evaluate this abnormality initiated by NP application.

There were some novel benefits to using the CatWalk method in the present study. First, it was possible to observe the behavioral change in the early phase using the CatWalk method. The mechanical withdrawal thresholds showed significant differences from days 14 to 21, but some parameters showed differences between the two experimental groups from day 7. Although some previous studies showed differences between the two groups on day 7, the weight of the rats and/or statistical methods differ from those of the present study. Thus, it is difficult to compare our results directly to those from previous studies. From this perspective, behavioral changes caused by pain can be effectively observed at an early time by gait analysis, and behavioral changes in the latter duration, which can indicate allodynia, can be efficiently revealed by measurement of withdrawal thresholds. In other words, the measurement of withdrawal thresholds using von Frey filaments has the advantage of sensitivity for detecting small changes in the NP group in latter time points and small changes in the sham group on day 7. However, the sensitivity was too high to identify differences between the two groups on day 7, while the specificity of gait analysis was high enough to detect differences between the two groups at early time points. For these reasons, not only behavioral testing, but also gait analysis should be used to evaluate pain behavior, especially at early time points.

Furthermore, that the pain due to NP could be evaluated by gait analysis is novel. In a clinical situation, diagnosis may be possible by gait analysis. Although it is indeed difficult to compare four-legged animals to humans, there have been several reports of gait analysis intended for evaluating humans with chronic low back pain (Kulkarni et al. 2005; Hamacher et al. 2014; Zahraee et al. 2014). One study reported that patients with chronic lower back pain had a long *stand* (Cimolin et al. 2011), which was similar to the results seen using the present rodent model. Since there are no data corresponding to various other gait parameters, additional gait examinations to connect the rodent model to humans with pain are needed.

There are some limitations of the CatWalk method. It is difficult to interpret the meaning of all gait parameters completely. The CatWalk method has many different parameters, and it is impossible to identify only one parameter that can indicate pain directly. Some studies reported the results of all parameters without describing the evaluation of each parameter (Encarnacion et al. 2011; Mead et al. 2011). However, for the purpose of evaluating pain using the CatWalk method, we should understand the definition of every parameter and determine the reason for each change comprehensively, such as pain that slows the gait pattern or causes the rat avoid touching the floor with its paw.

## Conclusions

In conclusion, rats with applied NP exhibited decreased withdrawal threshold and abnormal gait. Detailed behavioral evaluations of pain due to NP with gait analysis could be helpful for objective diagnosis. In the future, the interpfuretation of these parameters could be extended from in vivo studies to clinical situations.

## Abbreviations

CCD: Charge-coupled device
CCI: Chronic constriction injury
DRG: Dorsal root ganglion
LF: Left forepaw
LH: Left hind paw
NP: Nucleus pulposus
RF: Right forepaw
RH: Right hind paw
